# *Lactobacillus plantarum*-fermented persimmon juice alleviates alcohol-induced hepatic ferroptosis by activating the Keap1/Nrf2 antioxidant axis

**DOI:** 10.3389/fmicb.2026.1679233

**Published:** 2026-02-06

**Authors:** Huijuan Kuang, Qingyuan Ye, Juan Tong, Hong Fu, Zhiqiang Shi, Bin Zhu, Guotai Yang

**Affiliations:** 1 Department of Orthopedics, The First Affiliated Hospital of Xi’an Jiaotong University, Xi’an, China; 2 State Key Laboratory of Oral & Maxillofacial Reconstruction and Regeneration, National Clinical Research Center for Oral Diseases, Shaanxi Key Laboratory of Stomatology, Digital Dentistry Center, School of Stomatology, The Fourth Military Medical University, Xi’an, China; 3 State Key Laboratory of Holistic Integrative Management of Gastrointestinal Cancers, Department of Biochemistry and Molecular Biology, The Fourth Military Medical University, Xi’an, China; 4 State Key Laboratory of Oral & Maxillofacial Reconstruction and Regeneration, National Clinical Research Center for Oral Diseases, Shaanxi Clinical Research Center for Oral Disease, Department of Preventive Dentistry, School of Stomatology, The Fourth Military Medical University, Xi’an, China; 5 State Key Laboratory of Oral & Maxillofacial Reconstruction and Regeneration, National Clinical Research Center for Oral Diseases, Shaanxi Clinical Research Center for Oral Disease, Department of Medical Affairs, School of Stomatology, The Fourth Military Medical University, Xi’an, China; 6 Outpatient Department, General Hospital of the Xizang Military Command, Lhasa, China; 7 Center for Pharmaceutical Sciences, Faculty of Life Science and Technology, Kunming University of Science and Technology, Chenggong Campus, Kunming, China

**Keywords:** alcoholic liver disease, fermented persimmon juice, ferroptosis, Keap-1/Nrf2 signaling pathway, *Lactobacillus plantarum*

## Abstract

**Introduction:**

Alcoholic liver disease (ALD), induced by chronic and excessive alcohol consumption, poses a significant health risk, with higher female susceptibility. This study investigated Lactobacillus plantarum fermented persimmon juice (Fj) against ALD in female C57BL/6 mice.

**Methods:**

The model mice were orally treated with Fj or unfermented persimmon juice (Pj). The bioactive compound profiles of Fj and Pj were detected by HPLC-MS. The hepatoprotective effects was evaluated through assessments of hepatic lipid metabolism, Keap1/Nrf2 pathway proteins, and ferroptosis markers.

**Results:**

HPLC-MS analysis confirmed Fj was enriched in bioactive compounds including elevated antioxidants such as chlorogenic acid and p-hydrobenzoic acid. Daily Fj administration (10 μg/g BW) significantly improved hepatic lipid metabolism and regulated Keap1/Nrf2 signaling pathway, ultimately mitigating hepatic ferroptosis.

**Conclusion:**

These findings demonstrate that probiotic fermentation as a strategic approach to develop postbiotic-based functional beverages for mitigating alcohol-induced liver injury, offering translational potential against ALD progression.

## 1 Introduction

Alcoholic liver disease (ALD), induced by sustained and excessive alcohol consumption, has become one of the main risks to human health (Hyun et al., 2022; Mohajan, 2024). Hepatic steatosis is recognized as the earliest stage of ALD and occurs in more than 90% of individuals with alcoholism. Alcoholic liver steatosis may progress to steatohepatitis and cirrhosis, and eventually hepatocellular carcinoma (Alqahtani et al., 2023). Notably, alcohol consumption has become increasingly common among women facing challenging life circumstances and high-stress environments (Kersey et al., 2025; Kersey et al., 2022). Studies indicate that women exhibit more susceptibility to ALD compared to men (Ji et al., 2024). The observed gender disparity stems from higher blood alcohol concentrations in females at the equivalent alcohol intake (due to their lower average body weight), combined with reduced alcohol dehydrogenases (ADHs) content and activity, which impairs ethanol metabolism (Parlesak et al., 2002). Furthermore, chronic excessive alcohol consumption may trigger depression and other complications (Renu et al., 2023). Therefore, more attention should be given to alcohol-related health risks and complications in female populations.

Excessive lipid accumulation is a critical characteristic of hepatic steatosis in ALD (Huda et al., 2025; Malnick et al., 2022). Such abnormal lipid deposition potentially disrupts redox homeostasis and alters metabolic properties in the liver, eventually leading to hepatic lipid peroxidation (Soldo et al., 2022). Iron is essential for oxygen transport, cellular respiration, and DNA synthesis, all of which are related to lipid metabolism (Teh et al., 2024; Wu et al., 2023). Ferroptosis is regarded as an iron-dependent form of regulated cell death caused by unrestricted lipid peroxidation and subsequent membrane damage (Dixon and Olzmann, 2024). Specifically, an intracellularly damaged antioxidant system and iron overload can trigger the Fenton reaction, causing the accumulation of reactive oxygen species (ROS), the generation of lipid peroxides, and damage to the cell membrane structure (Leng et al., 2022; Wang et al., 2024). Hepatic iron overload has been detected in patients with advanced ALD and coexists with alcohol and ROS, consistent with other studies (Li et al., 2022; Li et al., 2023). Therefore, it is important to explore whether ferroptosis is involved in alcohol-induced hepatic steatosis and whether targeting ferroptosis can alleviate ALD.

Persimmon, a globally consumed fruit, is rich in nutrients such as carbohydrates, proteins, vitamin C, and amino acids. Persimmon is also an excellent source of bioactive compounds, including carotenoids and tannins (El Makhzangy et al., 2023). Studies have verified the anti-hangover effects of persimmon-related products, and persimmon extract has been shown to decrease triglyceride accumulation in hypertrophic adipocytes and improve liver damage (Zhou et al., 2019). However, the composition analysis indicated that persimmon contains a high level of sugar, which may aggravate fatty liver degeneration (Jiménez-Sánchez et al., 2015). Fermentation with beneficial bacteria offers a potential solution. The process not only improves the flavor of fruit and vegetable juice but also produces nutrients such as lactic acids and amino acids (Liu et al., 2024). The main benefit of fermented persimmon juice (Fj) lies in the conversion of saccharides to lactic acid by probiotics, thereby reducing the adverse effects of the high levels of saccharides in persimmon. Moreover, substances produced in probiotics are associated with enhanced anti-inflammatory and antioxidant activity. Therefore, we hypothesized that Fj may be effective in preventing the initiation and development of ALD, and the potential mechanism by which Fj alleviates alcohol-induced hepatic ferroptosis requires further exploration.

In this study, a female mouse model with alcoholic liver injury was established in accordance with the National Institute on Alcohol Abuse and Alcoholism (NIAAA) method. The study aimed to investigate alcohol-induced hepatic ferroptosis mediated by lipid peroxidation and to explore the mechanism of Fj in ALD. This research on the use of non-fermented persimmon juice (Pj) and the postbiotic preparation Fj in alleviating ALD provides a basis for studying the relationship between alcohol and ferroptosis in the liver. This study also offers insight into potential daily precautions for women to prevent the occurrence and development of ALD.

## 2 Materials and methods

### 2.1 Preparation of fermented persimmon juice

The most suitable endogenous strain, *Lactiplantibacillus plantarum* P202 (LP.P202), with excellent traits, was selected from persimmons to ferment persimmon juice. The optimized fermentation conditions were as follows: sterile persimmon juice (Pj) was supplemented with 4% glucose and 2% skimmed milk powder. A 5% inoculum of LP.P202 (pre-activated at 37 °C for 16 h twice) was then introduced into the mixture. Fermentation was carried out in an anaerobic incubator at 38 °C for 18 h. Following fermentation, Fj was sterilized by steam autoclaving at 100 °C for 3 min, and its pH was adjusted to 6.0 ± 0.5. The final product was then stored at 4 °C until use.

### 2.2 Animals administration

Seven-week-old female C57BL/6 mice were acclimatized for 7 days. The mice were kept at constant temperature and humidity with a 12-h light/dark cycle. The animal experiments were guided and supported by the laboratory animal care committee of Xi’an Jiaotong University.

After acclimatization, mice were randomly divided into four groups, with five mice in each group. All mice were adapted to the intake of Lieber DeCarli liquid diet for 5 days, and were then fed with 5% (v/v) alcohol for 10 days. Fermented persimmon juice or unfermented persimmon juice (10 μg/g body weight) was orally administered along with the alcohol treatment. On the last day of feeding, mice were orally administered with 35% (v/v) alcohol (5 g/kg BW) or maltose dextrin solution (9 g/kg body weight) as an isocaloric substitute. After 9 h, mice were weighed and then intraperitoneally injected with 40 mg/kg pentobarbital sodium for anesthetization. Blood was collected from the eyes after anesthesia, and all mice were euthanized by cervical dislocation. Subsequently, the thoracic cavity was cut, the liver was immediately isolated, weighed, and stored at −80 °C for subsequent experiments. Mice administered a normal liquid diet and phosphate-buffered saline (PBS) were considered controls.

### 2.3 Biochemical analysis

Liver tissues were weighed, homogenized in saline on ice, and then centrifuged at 2,500 rpm at 4 °C for 10 min, to obtain supernatant. Blood samples were centrifuged for 15 min at 3,000 × g under 4 °C to acquire serum. Alanine aminotransferase (ALT), aspartate aminotransferase (AST), total triglycerides (TG), total cholesterol (TC), glutathione (GSH), and malondialdehyde (MDA) were also evaluated using the ELISA kits (COIBO.BIO, Shanghai, China). All the above analyses were conducted in accordance with the kit’s instructions.

### 2.4 Hematoxylin and eosin staining (H&E)

Liver tissues were fixed in 4% formalin solution for 24 h, dehydrated with alcohol, immersed in wax, embedded, and sectioned. Next, the sections were stained with H&E and were observed under a microscope. The degree of liver injury was determined by histological scoring analysis. The assessment was expressed as the sum of the individual score grades of 0 (normal), 1 (mild injury), 2 (moderate injury), 3 (severe injury), and 4 (maximum injury) for each of the following three categories: inflammation infiltration, cytoplasm vacuolization, and nuclear condensation.

### 2.5 Oil red O staining

Liver tissue samples were fixed, dehydrated, embedded in OCT compound (Sakura, Tokyo, Japan), and sectioned into 10 μm frozen slices. The sections were stained with oil red O solution for 8–10 min and re-stained using Gill’s hematoxylin for 3–5 min. Finally, the sections were photographed under optical microscopes, and the area of the red-stained regions was quantified using ImageJ software (National Institutes of Health).

### 2.6 Inductively coupled plasma mass spectrometry (ICP-MS)

A 0.05–0.1 g portion of frozen liver samples were weighed, dissolved in HNO_3_/HClO_4_, and heated at 95 °C for 2 h until completely digested. The remaining solution was diluted with 25 mL of ultra-pure water. Inductively coupled plasma mass spectrometry was used to determine the total iron content in the liver.

### 2.7 Liquid chromatography-tandem quadrupole mass spectrometer (HPLC-MS)

The Pj or Fj supernatant was separated by centrifugation at 10,000 rpm/min for 15 min, filtered, and diluted to 10 mL.

The chromatographic process separation was performed using an Agilent SB-C18 column (4.6 mm × 250 mm, 5 μm) and maintained at 25 °C. The mobile phase comprised 0.1% formic acid solution (A) and acetonitrile solution (B). The elution gradient was as follows: 0–2 min, 5% B; 2–10 min, 5–20% B; 10–22 min, 20–40% B; 22–25 min, 40–70% B; 25–28 min, 70–90% B; 28–32 min, 90% B; and 32–37 min, 90–95% B. The flow rate was set at 0.3 mL/min, and the injection volume was 10 μL. The HPLC system was coupled to a 6530 tandem quadrupole-time-of-flight (Q-TOF) mass spectrometry equipped with an electrospray ionization (ESI) source, operating in negative ion mode. The conditions were as follows: capillary voltage, 4,000 V; nebulizer pressure, 35 psi; drying gas temperature, 350 °C; and drying gas flow rate, 12 L/min.

### 2.8 Dihydroethidium (DHE) staining

The fresh liver samples were collected and used to detect the ROS accumulation. All operations were performed according to the DHE staining guidelines provided by Servicebio Co., Ltd. (Wuhan, China), and quantified using ImageJ software (National Institutes of Health).

### 2.9 Immunohistochemistry (IHC)

The hepatic expressions of Keap1 and Nrf2 were detected using IHC. All procedures were performed according to the IHC guidelines of Servicebio Co., Ltd. (Wuhan, China), and quantified using ImageJ software (National Institutes of Health).

### 2.10 Real-time fluorescence quantitative PCR (RT-qPCR)

Total RNA was extracted from mouse liver tissue, and then the content of total RNA was measured and adjusted to the same concentration. The total RNA was reverse-transcribed to obtain cDNA for RT-qPCR. Table 1 indicates the primer sequences. All data were analyzed using the $2^{–\Delta \Delta C_{T}}$ method, with β-actin as the internal reference.

**Table 1:** Genes and primers selected for RT-qPCR.

Gene	Primer	Sequence (5′–3′)
*PPARα*	Forward	TACTGCCGTTTTCACAAGTGC
	Reverse	AGGTCGTGTTCACAGGTAAGA
*AMPK*	Forward	AGGCCCAAGATCCTCATGGA
	Reverse	GGGGGCTTTATCATTCGCTTC
*Fas*	Forward	AGGTGGTGATAGCCGGTATGT
	Reverse	TGGGTAATCCATAGAGCCCAG
*ACSL4*	Forward	GAAAGCAAACTGAAGGCGGC
	Reverse	TCACACTGGCCTGTCATTCC
*NOX1*	Forward	GTGCCGACAACAAGCTCAAA
	Reverse	ATGCTGCATGACCAGCAATG
*FTL*	Forward	CGTGGATCTGTGTCTTGCTTC
	Reverse	GTAGGAGCTAACCGCGAAGA
*FTH1*	Forward	CCCTTTGCAACTTCGTCGTTC
	Reverse	TTCAGAGCCACATCATCTCGG
*Keap1*	Forward	GAAGAGGCGGCAGAAGAAG
	Reverse	GCTCCAGGGCTATGACAGAT
*Nrf2*	Forward	TTCCATTTACGGAGACCCACC
	Reverse	GGATTCACGCATAGGAGCACTG
*CAT*	Forward	TTGTTCAGTGACCGAGGGATT
	Reverse	TTCCTGAGCAAGCCTTCCTG
*GSH*	Forward	ATCCCACTGCGCTCATGACC
	Reverse	AGCCAGCCATCACCAAGCC
*β-actin*	Forward	GCTCCTCCTGAGCGCAAGTA
	Reverse	CAGCTCAGTAACAGTCCGCC

### 2.11 Antioxidant activity determinations

For the ABTS assay, the procedure followed the method of Qie et al. (2023) with minor modifications. Stock solutions of 7.4 mM ABTS and 2.6 mM K_2_S_2_O_8_ were prepared. The working solution was prepared by mixing equal volumes of the two stock solutions and then maintained at room temperature in the dark for 12 h. Subsequently, the working solution was diluted with PBS before use to obtain an absorbance of 0.70 at 734 nm for the diluted ABTS radical solution. A 10-μL sample was combined with 2 mL diluted ABTS radical solution and incubated in the dark at room temperature before determining the absorbance at 734 nm using a spectrophotometer (PuXi Tonyong, China).

For the DPPH assay, the procedure followed the method of Qie et al. (2023) with minor modifications. The stock solution was prepared by dissolving 31.54 mg of DPPH in 200 mL of methanol and was stored at −20 °C until use. The sample (1.5 mL) was added to 1.5 mL of 0.4 mM DPPH reagent and incubated in the dark at room temperature for 30 min. The absorbance was measured at 517 nm.

For •OH assay, the procedure followed the method of Wen et al. (2023) with some modifications. Then, *o*-phenanthroline (1 mL, 2.5 mmol/L), PBS solution (1 mL, 0.01 mol/L, pH = 7.4), and FeSO_4_ (1 mL, 2.5 mmol/L) were fully mixed. To the obtained mixture, H_2_O_2_ (1 mL, 0.1%) and the sample (0.5 mL) were added. The mixture was then incubated at 37 °C for 1 h, after which the absorbance was determined at 536 nm.

For total antioxidant capacity (TAOC) assay, the kit was obtained from Nanjing Jiancheng Bioengineering Institute (Jiangsu, China). The sample (1.0 mL) was added into the reagent, and incubated at 37 °C for 10 min, after which the absorbance was determined at 529 nm.

### 2.12 Statistical analysis

In this experiment, graphs were generated by GraphPad Prism 8. All data are presented as means ± SD from at least three independent experiments. Statistical analyses were conducted using Student’s *t*-test (two-tailed) or one-way analysis of variance (ANOVA) with SPSS 19.0 (IBM, United States). For one-way ANOVA, Tukey’s *post-hoc* test was applied for multiple comparisons between the groups. Differences were considered statistically significant at ^*^p < 0.05 and highly significant at ^**^p < 0.01.

## 3 Results

### 3.1 Oral administration of Pj or Fj ameliorates alcohol-induced liver damage

**Figure 1:**
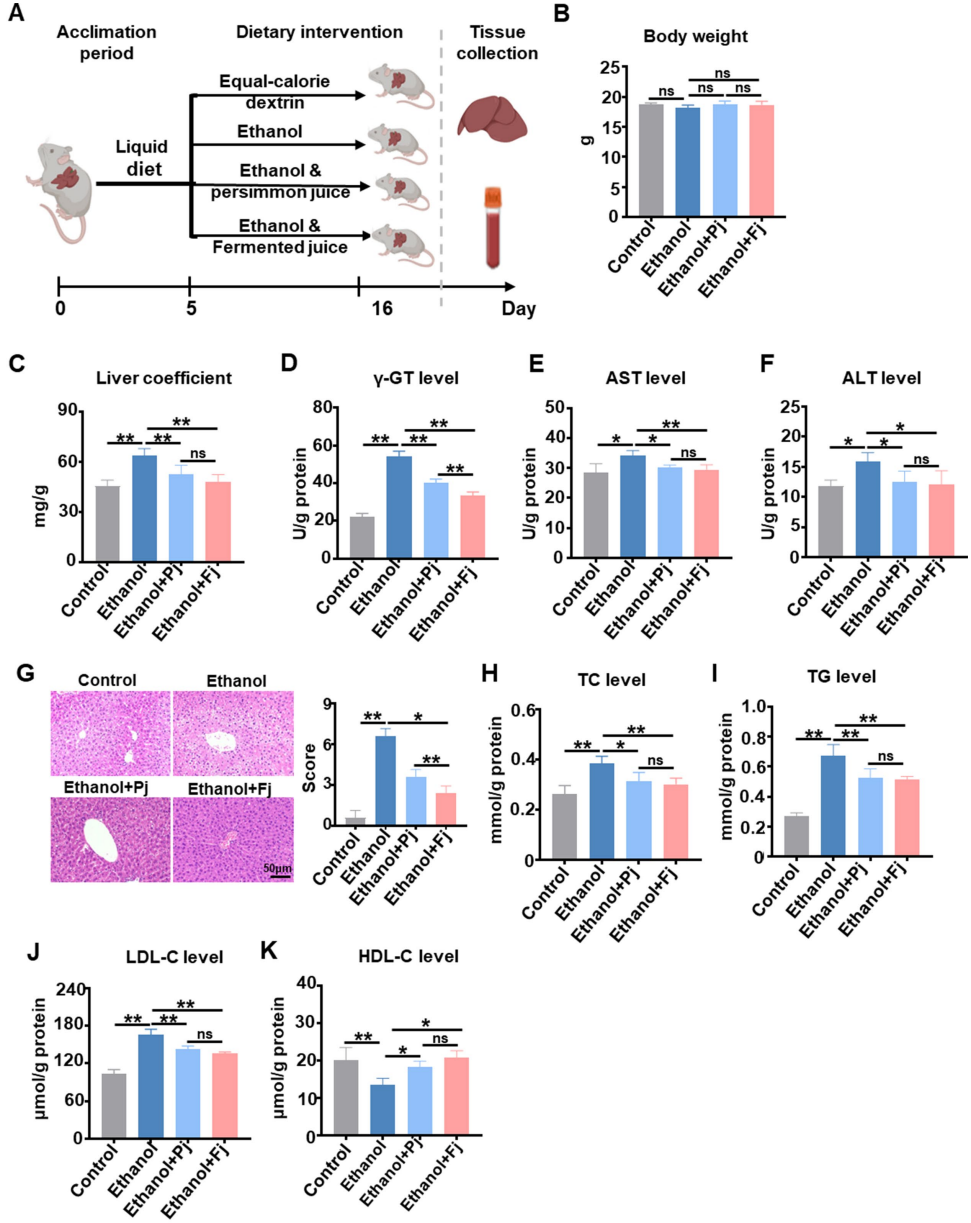
Fermented persimmon juice alleviated alcohol-induced liver damage. **(A)** The experiment scheme. The images created with BioRender.com with permission. **(B)** Body weight (*n* = 5 mice). **(C)** Liver organ coefficient (*n* = 5 mice). **(D)** γ-Glutamyl transpeptidase level (*n* = 5 mice). **(E)** Serum ALT level (*n* = 5 mice). **(F)** Serum AST level (*n* = 5 mice). **(G)** Liver sections with H&E staining and quantitative analysis of histological scores, the scale bar is 50 μm, *n* = 5 mice. **(H)** TC level. **(I)** TG level. **(J)** HDL-C level. **(K)** LDL-C level. Data are represented as means ± SD. **(B–K)** Statistical significance was assessed by one-way ANOVA with Tukey’s *post-hoc* test. ns, not significant; ^*^p < 0.05 and ^**^p < 0.01.

Following alcohol-induced liver damage, mice were orally administered with Pj or Fj to evaluate their therapeutic efficacy (Figure 1A). At the end of the trial, the mice in all groups showed no significant difference in body weights (Figure 1B), while the liver organ coefficient of ethanol + Fj group was significantly lower than the ethanol or ethanol + Pj group (Figure 1C), suggesting that Fj may more effectively attenuate alcohol-induced hepatic hypertrophy. Consistent with the alcohol-induced liver injury model, the alcohol consumption significantly elevated the hepatic γ-glutamyl transferase (γ-GT), AST, and ALT levels compared with the control group. Importantly, Pj and Fj treatment effectively reversed these elevations, bringing the levels closer to those of the control group (Figures 1D–F). Histopathological examination through H&E staining provided further evidence. As shown in Figure 1G, the liver of alcohol-treated mice showed hepatocyte steatosis, accompanied by severe inflammatory cell infiltration and hepatic cellular necrosis. However, Pj or Fj treatment alleviated these histopathological lesions, with a more pronounced effect observed in the Fj group (Figure 1G).

Ingested alcohol is primarily metabolized in the liver and induces hepatic triglyceride accumulation (Zhang et al., 2025; De Bruyne et al., 2017). Therefore, hepatic levels of TC, TG, HDL-C, and LDL-C were examined. Compared to the control group, the ethanol group showed higher hepatic TC, TG, and LDL-C levels, while HDL-C levels were significantly lower (Figures 1H–K). Pj or Fj treatment significantly improved these adverse alterations (Figures 1H–K). The biochemical and morphological analyses strongly suggest that Pj and Fj hold promise as a therapeutic agent for mitigating alcohol-induced liver injury, warranting further investigation into their underlying mechanisms.

### 3.2 Fj reduces hepatic lipid accumulation and oxidative stress through the AMPK-PPARα signaling pathway

**Figure 2:**
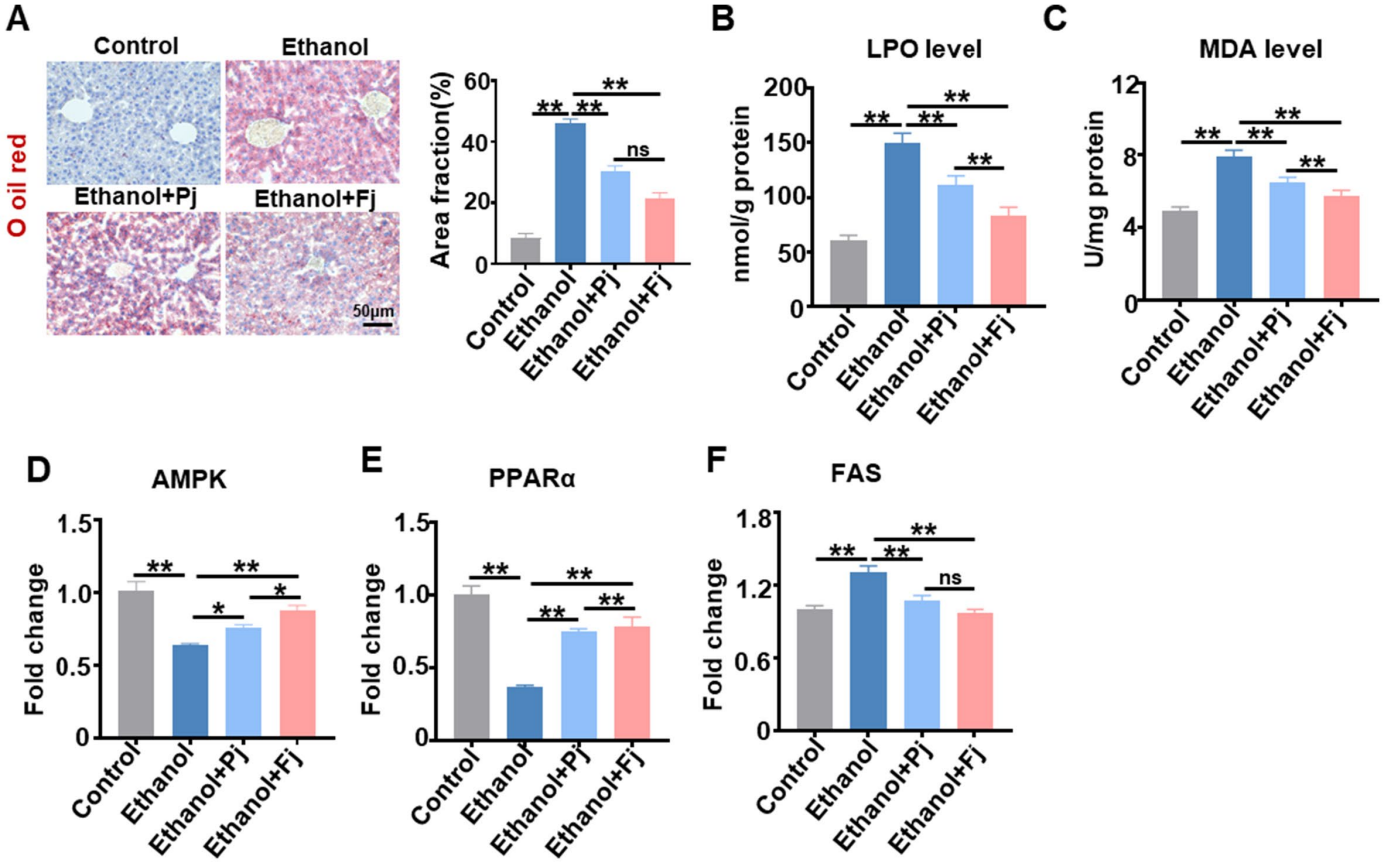
Fermented persimmon juice alleviates alcohol-induced severe lipid peroxidation and lipid metabolism disorders induced by consumption. **(A)** Representative images of the liver with oil red O staining and quantification of oil red O staining area; the scale bar is 50 μm, *n* = 5 mice. **(B)** Lipid-peroxides and **(C)** malonaldehyde level, *n* = 5 mice. Lipid metabolism-related genes **(D)** *PPARα* **(E)** *AMPK* **(F)** *FAS* levels, *n* = 5 mice. Data are represented as means ± SD. **(A–F)** Statistical significance was assessed by one-way ANOVA with Tukey’s *post-hoc* test. ^*^p < 0.05 and ^**^p < 0.01.

Based on the aforementioned alterations in lipid metabolism, the oil red O staining was further employed to assess the hepatic lipid accumulation. The oil red O specifically stains neutral triglycerides, lipids, and lipoproteins in tissues and cells (Ciociola et al., 2024). As shown in Figure 2A, alcohol consumption induced marked lipid accumulation in the liver, which was significantly attenuated by oral administration of Pj and Fj, with a more pronounced effect observed in the Fj group. Quantification of the oil red O-stained area fraction confirmed these histological observations (Figure 2A). To explore the underlying mechanism, we examined the alcohol-induced oxidative stress and lipid peroxidation. Chronic alcohol metabolism generates excessive oxygen radicals, leading to hepatic lipid peroxidation (Contreras-Zentella et al., 2022). As key indicators of lipid peroxidation, the levels of lipid peroxide (LPO) and malondialdehyde (MDA) were increased in the ethanol group. Both Pj and Fj treatment effectively suppressed these increases, with Fj treatment demonstrating superior efficacy (Figures 2B,C). These results suggest that alleviation of oxidative damage may represent an important pathway through which Fj ameliorates alcohol-induced hepatic lipid metabolic disorders.

To further elucidate the molecular mechanisms involved in lipid regulation, we examined the expression of key genes in the AMPK-PPARα signaling pathway, which governs fatty acid β-oxidation (Dong et al., 2024). Ethanol exposure suppressed *AMPK* and PPARα expression, while increasing FAS expression. Notably, Fj treatment reversed these aberrant patterns, restoring them to near-normal levels (Figures 2D–F). These findings are consistent with the histological and biochemical improvements, suggesting that Fj may mitigate alcohol-induced hepatic lipid metabolic disorders through the AMPK-PPARα pathway, promote fatty acid oxidation, and suppress lipid synthesis.

### 3.3 Fj alleviates alcohol-induced liver injury by suppressing ferroptosis

**Figure 3:**
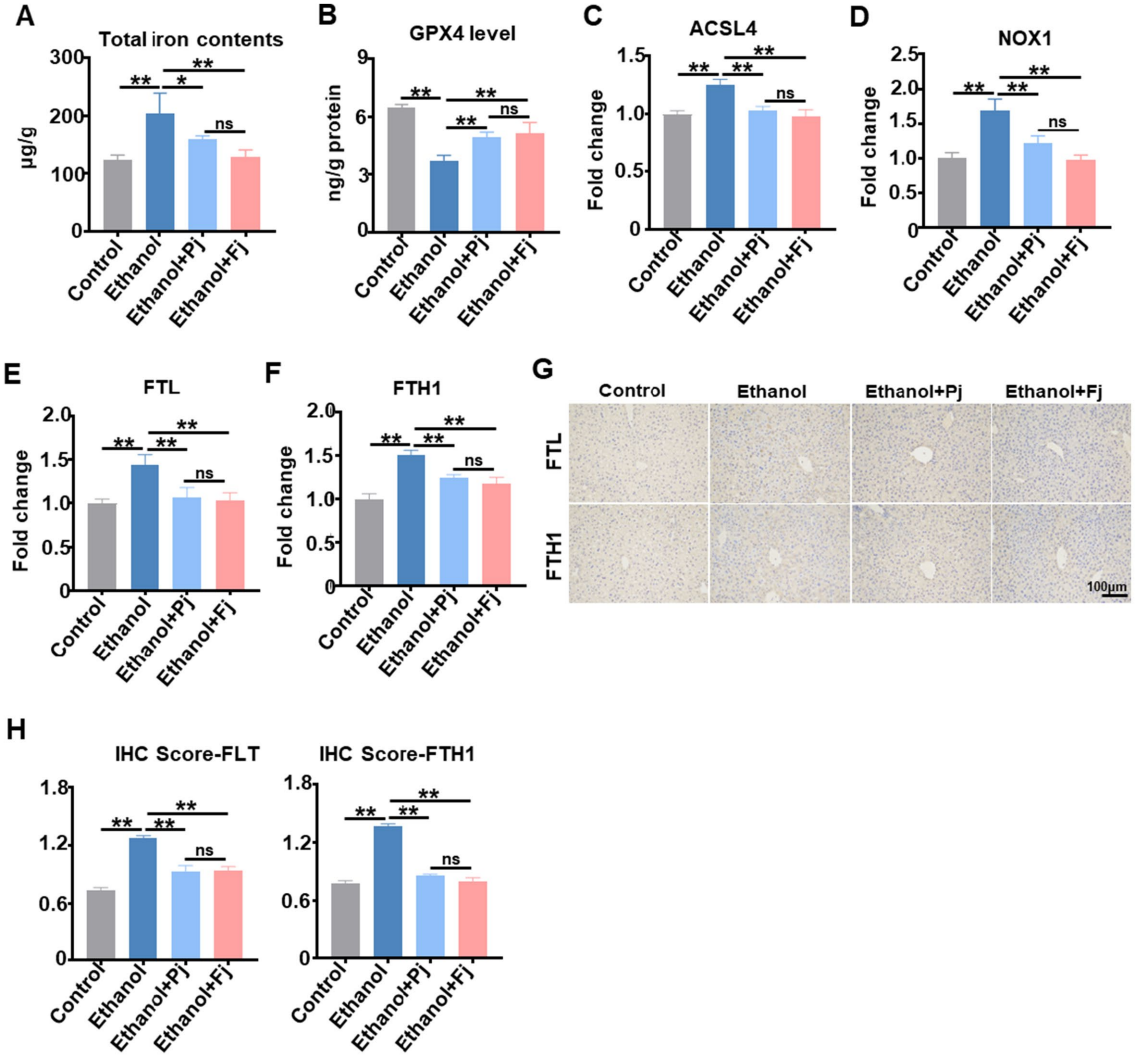
Fermented persimmon juice alleviates alcohol-induced hepatic ferroptosis. **(A)** The content of iron in the liver, *n* = 5 mice. **(B)** The hepatic levels of GPX4, *n* = 5 mice. **(C–F)** Ferroptosis-related genes of **(C)** ACSL4, **(D)** NOX1, **(E)** FTL, and **(F)** FTH1 levels, *n* = 5 mice. **(G)** Protein level of FTL and FTH in the liver. The scale bar is 100 m, *n* = 5 mice. **(H)** Quantification of immune-expression of FTL and FTH, *n* = 5 mice. Data are represented as means ± SD. **(A–F,H)** Statistical significance was assessed by one-way ANOVA with Tukey’s *post-hoc* test. ^*^p < 0.05 and ^**^p < 0.01.

Accumulation of lipid peroxides is a critical trigger of ferroptosis. ICP-MS was used to quantify hepatic total iron levels (Figure 3A). Alcohol consumption significantly increased hepatic total iron content, whereas Pj treatment effectively restored iron concentrations to near-baseline levels, with Fj administration showing a particularly strong restorative effect (Figure 3A). Glutathione (GSH) peroxidase 4 (GPX4), an enzyme that inhibits iron-dependent lipid peroxidation, was significantly reduced in the ethanol group (Figure 3B). In contrast, Pj and Fj treatment increased GPX4 levels, with Fj treatment demonstrating greater restoration (Figure 3B). We then measured the expression of ferroptosis-related genes (ACSL4 and NOX1) and ferritin subunits (FTL and FTH1). As expected, alcohol consumption significantly increased the expression of these genes, while Fj treatment induced greater restoration (Figures 3C–F). Consistent with the transcriptional changes, IHC staining confirmed the protein expression levels of FTL and FTH1 across groups (Figures 3G,H). These results collectively indicate that alcohol consumption induces iron overload and dysregulates ferroptosis-related markers, whereas Fj treatment effectively counteracts these abnormalities. Fj may mitigate alcohol-induced liver injury, at least in part, by regulating iron homeostasis and suppressing ferroptosis.

### 3.4 Fermentation enhances the antioxidant capacity and alters the metabolite profile of Pj

**Figure 4:**
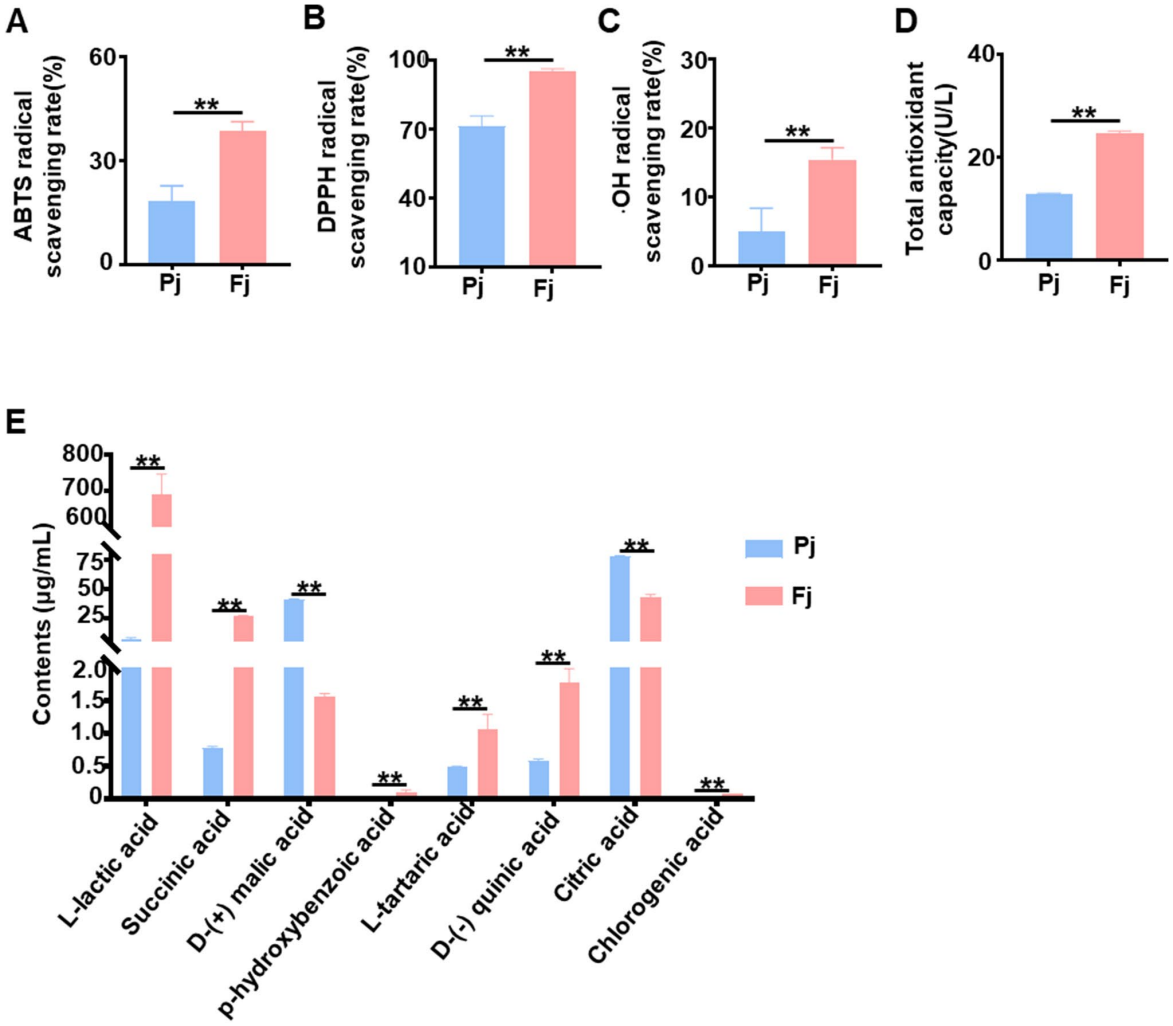
Fermentation alters the antioxidant activity and metabolite profile of Pj and Fj. **(A–C)** ABTS, DPPH, and OH radical scavenging rates, *n* = 3 biologically independent samples. **(D)** TAOC levels, *n* = 3 biologically independent samples. **(E)** Key differential substance in fermented persimmon juice analyzed by HPLS-MS, *n* = 3 biologically independent samples. Data are represented as means ± SD. **(A–E)** Statistical significance was assessed using one-way ANOVA with Tukey’s *post-hoc* test. ^*^p < 0.05 and ^**^p < 0.01.

Previous studies have reported that the fermentation by *Lactobacillus* may increase amino acids, short-chain fatty acids, and other nutrients in both fruits and vegetables (Han et al., 2025). Notably, the flavonoids and polyphenols present in fermented products act as potent scavengers of oxygen-free radicals. To examine the effect of fermentation on the antioxidant properties of Pj, we assessed its capacity to scavenge ABTS, DPPH, OH, and TAOC *in vitro*. As shown in Figures 4A–D, fermentation with *L. plantarum* P202 significantly enhanced the antioxidant capacity of Pj, indicating its potential to improve hepatic antioxidant capacity. Furthermore, HPLC-MS analysis was employed to evaluate the compositional changes in Pj before and after fermentation. And the results revealed significant increases in L-lactic acid, succinic acid, L-tartaric acid, D-(−) quinic acid, and chlorogenic acid, whereas the contents of D-(+) malic acid and citric acid levels significantly decreased. Furthermore, a newly detected metabolite, p-hydroxybenzoic acid, was produced during the fermentation process (Figure 4E). These compositional shifts may contribute to the enhanced bioactivity of Fj, particularly its improved antioxidant and hepatoprotective effects.

### 3.5 Fj enhances hepatic antioxidant capacity by scavenging ROS and restoring GSH/GSH-Px

**Figure 5:**
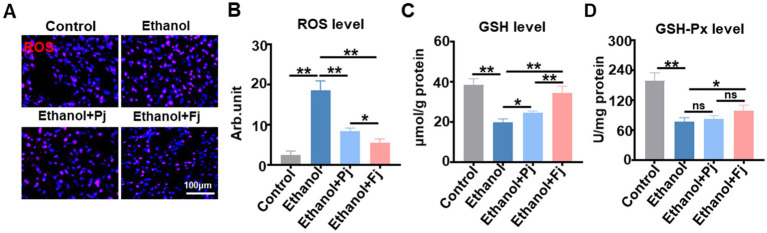
Fermented persimmon juice attenuates alcohol-induced oxidative stress. **(A)** Image of ROS in the liver and the quantitative measurement of ROS levels, the scale bar is 100 μm, *n* = 5 mice. **(B)** Glutathione level, *n* = 5 mice. **(C)** Glutathione peroxidase level, *n* = 5 mice. Data are represented as mean ± SD. **(A–C)** Statistical significance was assessed by one-way ANOVA with Tukey’s *post-hoc* test. ^*^p < 0.05 and ^**^p < 0.01.

To evaluate the effect of Fj on hepatic antioxidant capacity, DHE staining was employed to assess ROS generation. As shown in Figure 5A, alcohol consumption induced a marked increase in red fluorescence, indicative of elevated ROS levels, which was suppressed by Fj treatment. Semi-quantitative analysis of ROS content further confirmed these observations (Figure 5B). As the clearance of excess ROS was regulated by endogenous antioxidants, the hepatic levels of GSH and GSH-Px were measured. The results indicated that alcohol consumption depleted both GSH and GSH-Px levels; conversely, Pj and Fj treatment restored their levels, with Fj showing a more substantial recovery, elevating GSH and GSH-Px levels by 9.6 and 29.8%, respectively (Figures 5C,D). These findings indicated that Fj potently enhances the hepatic antioxidant defense system, contributing to the hepatoprotective effect.

### 3.6 Fj activates the Keap1-Nrf2 signaling pathway to alleviate alcohol-induced ferroptosis

**Figure 6:**
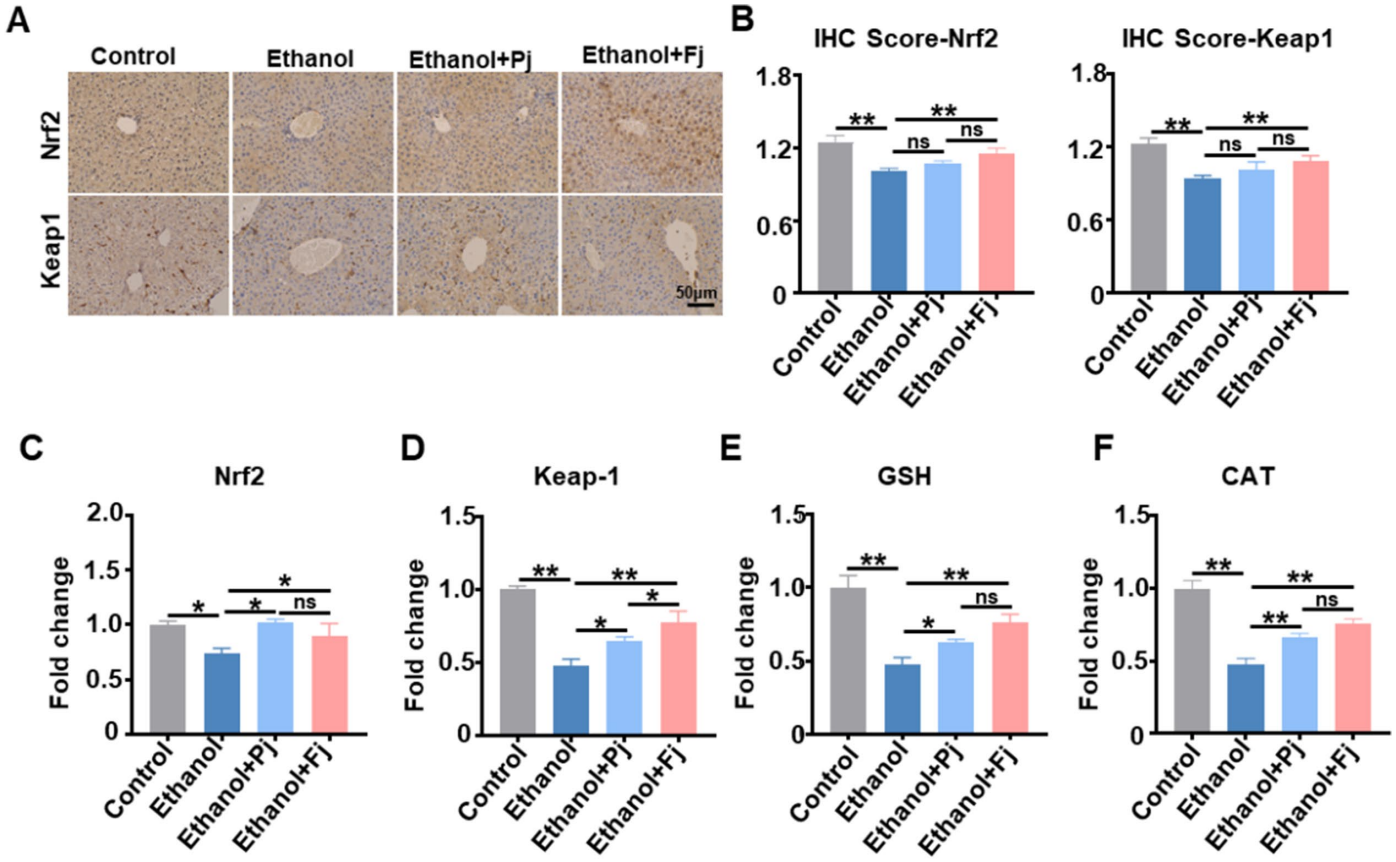
Fermented persimmon juice upregulated the Keap1-Nrf2 axis and its downstream antioxidant genes. **(A)** Representative images of liver sections with IHC staining to examine the protein levels of Keap1 and Nrf2, scale bar is 100 μm. **(B)** The IHC score of Keap1 and Nrf2, *n* = 5 mice. **(C–F)** Hepatic expression of Keap1-Nrf2-ARE signaling pathway: **(C)** Nrf2, **(D)** Keap1, **(E)** CAT, and **(F)** GSH levels. Data are represented as means ± SD. **(B–F)** Statistical significance was assessed by one-way ANOVA with Tukey’s *post-hoc* test. ^*^p < 0.05 and ^**^p < 0.01.

Inactivation of GPX4 and depletion of GSH are recognized as pivotal events driving lipid peroxidation during ferroptosis (Liu et al., 2023). Given the enhanced antioxidant capacity and ROS-scavenging activity observed with Fj treatment, we hypothesized that Fj alleviates alcohol-induced ferroptosis through modulation of oxidative stress-related signaling pathways. We therefore focused on the Keap1-Nrf2 pathway, a central regulator of antioxidant responses (Chen et al., 2024; Liu et al., 2022). As shown in Figures 6A,B, IHC quantification indicated that alcohol consumption suppressed hepatic protein levels of Keap1 and Nrf2. In contrast, Fj administration significantly increased the hepatic protein expression levels, suggesting activation of this cytoprotective pathway. RT-qPCR analysis revealed that ethanol exposure downregulated hepatic mRNA levels of Keap1, Nrf2, and the downstream antioxidants GSH and CAT, whereas Fj treatment restored their expression (Figures 6C–F). Although the apparent decrease in total Keap1 and Nrf2 protein detected by IHC merits further investigation, the consistent upregulation of their mRNA and key downstream targets supports the conclusion that Fj activates the Nrf2-mediated cytoprotective program, thereby reinforcing cellular resistance to ferroptosis.

This mechanistic insight suggests that Fj mitigates alcohol-induced ferroptosis not only by directly scavenging ROS but also by reinforcing the endogenous antioxidant defense system.

## 4 Discussion

**Figure 7:**
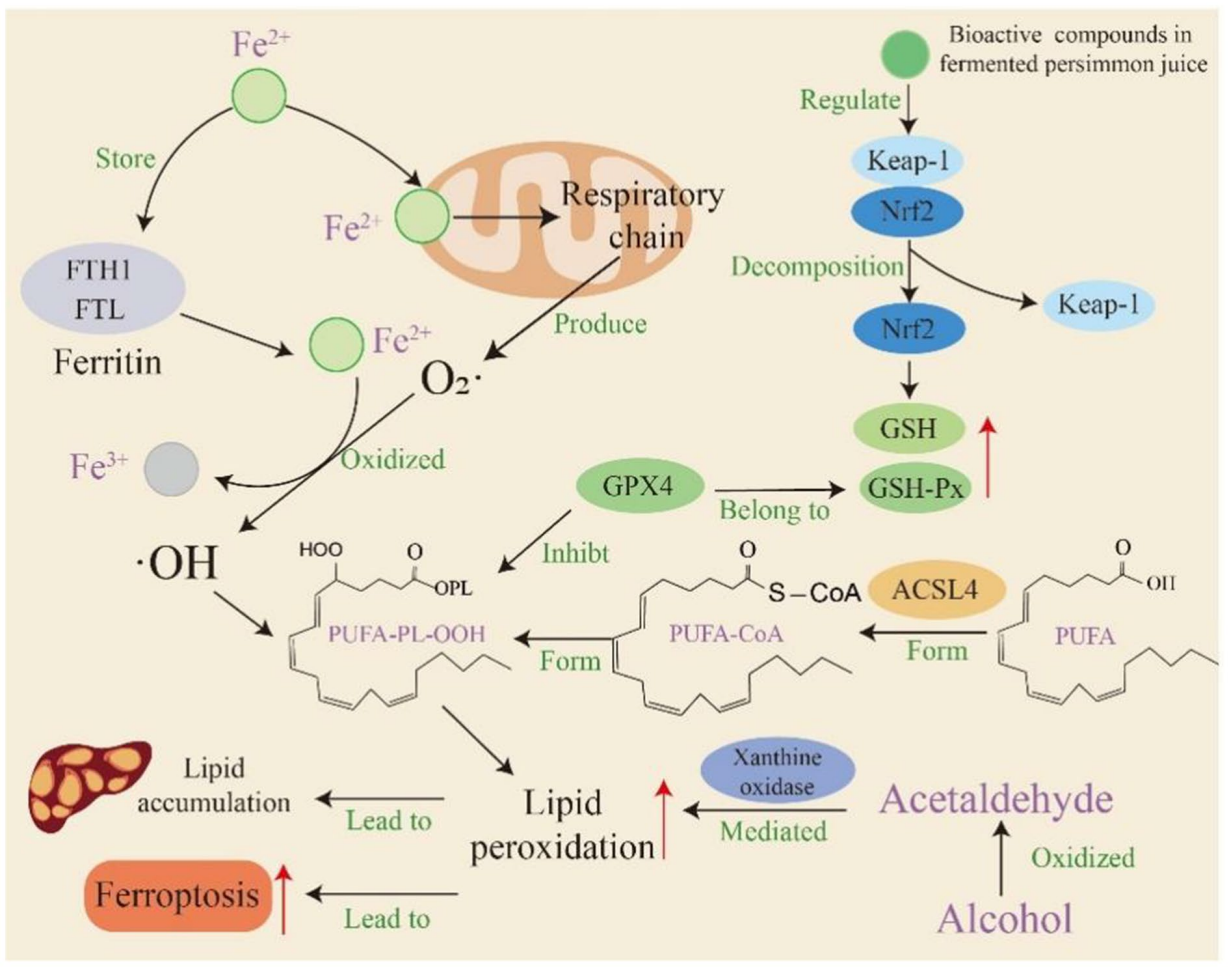
Fermented persimmon juice alleviates ferroptosis mediated by lipid peroxidation in alcoholic fatty liver by enhancing antioxidant capacity and regulating Keap1/Nrf2 signaling pathway.

The rising social acceptance of alcohol consumption, particularly among women, highlights the urgent need for effective interventions against alcohol-related liver diseases (Otto, 2024). Our study reveals that Fj (persimmon juice fermented with *Lactiplantibacillus plantarum* P202) protects against alcohol-induced liver injury in a female mouse model and exhibits superior efficacy compared to non-fermented persimmon juice. The protective effects of Fj were evidenced by improved serum biomarkers, restored lipid homeostasis, improved histopathological injury, and attenuated hepatic steatosis. More importantly, we provide evidence that Fj alleviates ALD by enhancing antioxidant capacity and suppressing ferroptosis through activation of the Keap1-Nrf2 pathway, extending beyond previously reported benefits of persimmon products (Figure 7).

Consistent with prior studies on hypolipidemic properties of persimmon (Pérez-Piñero et al., 2024; Wang et al., 2023), Fj administration significantly reduced hepatic TC, TG, and LDL-C levels while increasing HDL-C. Our key novelty is the direct comparison between Fj and Pj. The comparison clearly showed that fermentation enhances these lipid-regulating effects. The reduction in lipid droplets and liver coefficient in the Fj group aligns with reports that fermented fruit products promote lipid oxidation and reduce adiposity (Omary et al., 2025; Xiao et al., 2023). This study proposed that the fermentation-induced enrichment of organic acids, such as lactic and succinic acid, may be a key factor that activates hepatic AMPK-PPARα signaling, a pathway that plays a critical role in regulating β-oxidation and fatty acid transport (Yang et al., 2024; Zhang et al., 2023).

A key advance of this study is the identification of ferroptosis as a target of Fj in ALD. While earlier reports linked persimmon extracts to antioxidant effects (Choudhary et al., 2023), few connected these effects to iron-dependent cell death regulation. Our data showed that alcohol feeding led to iron overload, GPX4 downregulation, and elevated ACSL4 (all established hallmarks of ferroptosis) (Liu et al., 2025), which have recently been implicated in ALD pathogenesis. Fj treatment counteracted these changes more effectively than Pj, suggesting that fermentation enhanced the bioactive compounds responsible for iron homeostasis and anti-lipoperoxidant activity. This finding supports Fj as a potential ferroptosis inhibitor, with the potential applications in metabolic liver disease research.

The enhanced efficacy of Fj is consistent with reports that lactic acid fermentation boosts the antioxidant profile of fruits (Bryukhanov et al., 2022; Khubber et al., 2022). Using HPLC-MS, we confirmed increases in chlorogenic acid, tartaric acid, and newly generated p-hydroxybenzoic acid-phenolics—known for radical-scavenging activities. *In vivo*, Fj reduced ROS and restored GSH and GSH-Px, consistent with studies on probiotic-fermented foods ameliorating oxidative stress (Ibrahim et al., 2023; Liu et al., 2024). Importantly, we further delineated the upstream mechanism: Fj activates the Keap1-Nrf2 pathway, leading to upregulation of cytoprotective genes. This aligns with reports that Nrf2 activation mitigates both oxidative stress and ferroptosis (Khan et al., 2024; Yan et al., 2023), providing a unified mechanistic explanation for Fj’s multi-level protection.

It is important to note that a limitation of our study is the absence of a positive control using silymarin. Compared with silymarin (an herbal agent used for protecting the liver from various chemicals or toxins, including alcohol) (Wadhwa et al., 2022), Fj demonstrates a unique multi-target mechanism of action. Previous studies have confirmed that silymarin primarily exerts antioxidant and anti-inflammatory effects through its flavonolignan components (Shivaprasad et al., 2025; Song et al., 2006), alleviating oxidative stress and hepatic steatosis in alcohol-induced liver injury models. However, our findings reveal that Fj not only exhibits comparable antioxidant capacity but also provides more comprehensive protection by regulating lipid metabolic homeostasis and suppressing ferroptosis. In particular, Fj activated the AMPK-PPARα pathway and restored the GPX4 and GSH systems, which are broader than those of silymarin. Furthermore, the fermentation process produced unique metabolites such as *p*-hydroxybenzoic acid. These special substances may endow Fj with distinct bioactivities.

While our study establishes Fj’s benefits in female mice—a population with higher susceptibility to ALD—it remains unclear whether the effects are sex-specific. Future studies should include male cohorts to assess generalizability. In addition, although we identified several fermentation-induced metabolites, pinpointing the exact bioactive components responsible for the observed effects will require further fractionation and dose–response experiments.

## 5 Conclusion

This study demonstrated that Fj alleviates alcohol-induced disruption of lipid metabolism and ferroptosis by regulating the Keap1/Nrf2 signaling pathway. Moreover, this study provides a theoretical foundation for postbiotic-based interventions to alleviate liver damage caused by chronic alcohol consumption and prevent ALD progression. Furthermore, probiotic fermentation is an effective method for enhancing the therapeutic potential of foods/diets.

## Data Availability

The datasets presented in this study can be found in online repositories. The names of the repository/repositories and accession number(s) can be found in the article/supplementary material.
